# Impaired Human Papillomavirus (HPV) Clearance in a Patient on Upadacitinib for Atopic Dermatitis: A Case of Refractory Genital Warts Resolved After Drug Withdrawal

**DOI:** 10.7759/cureus.97974

**Published:** 2025-11-27

**Authors:** Norifumi Kennoki, Kyotaro Nohata, Yuta Nakamori

**Affiliations:** 1 Sexually Transmitted Infections, Ginza Hikari Clinic, Tokyo, JPN

**Keywords:** atopic dermatitis, cellular immunity, condyloma acuminatum, genital warts, hpv clearance, human papilloma virus, imiquimod, immunosuppression, janus kinase 1 inhibitor, upadacitinib

## Abstract

We report a case of refractory condyloma acuminatum in a young woman receiving upadacitinib for moderate-to-severe atopic dermatitis. The patient developed widespread genital and perianal warts while on upadacitinib, a selective Janus kinase 1 (JAK1) inhibitor. Despite appropriate topical treatment with imiquimod, adjunctive cryotherapy, and herbal immunomodulatory agents, the lesions remained resistant to therapy. After consultation with the prescribing dermatologist, upadacitinib was discontinued while imiquimod was continued as monotherapy. Remarkably, within several weeks of drug cessation, the lesions regressed, and complete clinical resolution was observed within three months. No recurrence has been noted in the 10 months following clearance. This case underscores the potential for JAK1 inhibition to impair cellular antiviral immunity, particularly in the context of human papillomavirus (HPV)-related diseases such as condyloma acuminatum. The immunologic mechanism may involve suppression of interferon-γ, interleukin-2, and other cytokine pathways critical to viral clearance. Clinicians should be aware of this possible adverse interaction, especially when prescribing systemic immunomodulatory agents to sexually active patients or those with a history of HPV infection. Consideration of genital examination and prophylactic HPV vaccination prior to initiating JAK inhibitor therapy may be warranted. Furthermore, in cases of treatment-resistant genital warts, reassessing the patient’s systemic immune status and current immunosuppressive medications may lead to improved clinical outcomes.

## Introduction

Anogenital warts are caused in approximately 90% of cases by non-oncogenic human papillomavirus (HPV) types 6 or 11 [1]. HPV replicates in stratified epithelium by infecting basal keratinocytes and coordinating their life cycle with epithelial differentiation, enabling viral genome amplification and assembly of infectious particles [2].

In immunocompetent, HIV-negative adults with anogenital warts, topical 5% imiquimod applied by patients has demonstrated complete clearance in roughly 40-50% of cases within 16 weeks in randomized, vehicle-controlled studies [3]. In comparison, provider-administered cryotherapy with liquid nitrogen has shown even higher short-term clearance rates, about 87% at three months, in a head-to-head clinical trial [4], supporting the overall effectiveness of both modalities in immunocompetent individuals.

In comparison with immunocompetent individuals, the efficacy of both patient-applied therapies, such as 5% imiquimod and provider-administered destructive procedures, including cryotherapy, electrocautery, or surgical excision, appears diminished among immunosuppressed patients. A systematic review by Moore et al. noted that clearance rates achieved in randomized trials among immunocompetent participants were not reproduced in HIV-positive cohorts, where lower response and higher recurrence were observed [5]. Similarly, Kwak et al. described that solid-organ transplant recipients under chronic immunosuppression often present with extensive, treatment-refractory anogenital warts despite repeated sessions of cryotherapy or surgical removal, indicating that impaired cell-mediated immunity compromises treatment outcomes and favors recurrence [6].

Upadacitinib, a selective Janus kinase 1 (JAK1) inhibitor, has been approved for the treatment of moderate-to-severe atopic dermatitis based on phase 3 randomized trials demonstrating significant clinical improvement in disease severity and pruritus compared with placebo [7].

The association between JAK inhibition and herpes zoster is well established, with network meta-analyses of randomized trials showing higher zoster incidence for several JAK inhibitors compared with placebo (notably peficitinib, baricitinib, and upadacitinib) in immune-mediated diseases [8]. By contrast, the potential impact of JAK blockade on HPV control is less recognized.

## Case presentation

**Figure 1:**
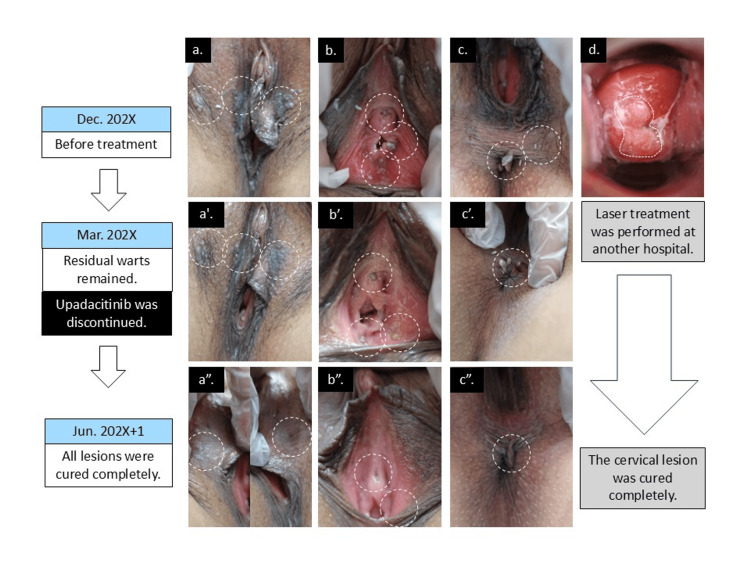
Clinical course and response to treatment (a–c) Genital and perianal condyloma acuminatum lesions before intervention (Dec. 202X). (a′–c′) After three months of treatment with imiquimod, cryotherapy, and coix seed extract, partial regression was observed, but residual warts remained. Upadacitinib was discontinued (Mar. 202X). (a″–c″) Complete resolution of lesions was achieved three months later with continued imiquimod monotherapy (Jun. 202X+1). (d) Cervical lesions were detected at initial presentation and treated with CO₂ laser therapy at another hospital. Follow-up confirmed complete resolution of cervical lesions. Original illustration created by the authors (mechanisms referenced in the text).

A woman in her 20s presented with a two-month history of gradually enlarging papular lesions on the external genitalia. She had a history of atopic dermatitis since childhood, for which she had been receiving upadacitinib. She had started oral upadacitinib 15 mg daily approximately seven months before presentation, and due to insufficient improvement of her atopic dermatitis symptoms, the dose had subsequently been increased to 30 mg daily about one month prior to her first visit. Her dermatitis was well controlled with this treatment. She reported no other significant medical history, although she had been previously diagnosed and treated for *Candida spp.*, *Trichomonas vaginalis*, *Chlamydia trachomatis*, and *Neisseria gonorrhoeae* infections. She reported recent sexual contact with three different partners within the past several months. On examination, multiple verrucous papules were noted on the vulva, perianal region, vaginal introitus, and periurethral area, consistent with condyloma acuminatum (Figure 1a-Figure 1c). In addition, nodular lesions on the cervical epithelium were detected on speculum examination (Figure 1d). Based on these findings, cervical condyloma was diagnosed, and the patient was referred to a gynecologist, where she subsequently underwent carbon dioxide laser ablation for the cervical lesion. At our clinic, topical imiquimod 5% cream was initiated three times per week. Oral coix seed extract and weekly cryotherapy were also added to the regimen. After three months of this combination therapy, some reduction in lesion size was observed; however, residual warts remained, and new lesions continued to appear (Figure 1a’-Figure 1c'). The patient adhered well to the treatment. Given the suboptimal therapeutic response and considering the potential immunosuppressive effects of JAK1 inhibition on antiviral immunity, we consulted with the patient's prescribing dermatologist. Upadacitinib was then discontinued, and topical imiquimod was continued as monotherapy. Four weeks after cessation of upadacitinib, marked regression of the genital lesions was observed. After an additional three months of continued topical therapy, the lesions had completely resolved (Figure 1a’’-Figure 1c”). At the time of this writing, 10 months have passed since clearance of the warts, and no recurrence has been observed.

## Discussion

JAK inhibitors, including upadacitinib, disrupt intracellular signaling downstream of cytokines such as interferon-γ and interleukin-2, which are pivotal for T-cell-mediated antiviral immunity [9,10]. By blocking JAK1-dependent STAT activation, these agents can attenuate interferon responses that normally restrict viral replication and promote clearance of infected keratinocytes. As such, prolonged JAK inhibition may impair host defense against HPV, potentially contributing to viral persistence or progression of HPV-related disease.

While the increased risk of herpes zoster and other opportunistic infections during JAK inhibitor therapy is well established [8], their influence on HPV-associated conditions has received little attention. Seale et al. recently described a case of refractory flat warts in a patient with atopic dermatitis shortly after initiating upadacitinib therapy, suggesting that JAK1 blockade may exacerbate HPV-driven cutaneous lesions [11]. Likewise, Wu et al. reported eruptive condyloma acuminatum following infliximab initiation, emphasizing that immune suppression from various biologic agents can alter HPV pathogenesis [12].

In our patient, combination therapy with imiquimod, cryotherapy, and coix seed extract was ineffective while receiving upadacitinib, despite excellent adherence. Strikingly, the warts regressed rapidly after discontinuation of upadacitinib, responding well to imiquimod monotherapy. This temporal relationship supports the notion that systemic immunosuppression induced by JAK1 inhibition may diminish treatment responsiveness and promote persistence of anogenital warts, paralleling findings observed in other immunocompromised populations [5,6].

**Figure 2:**
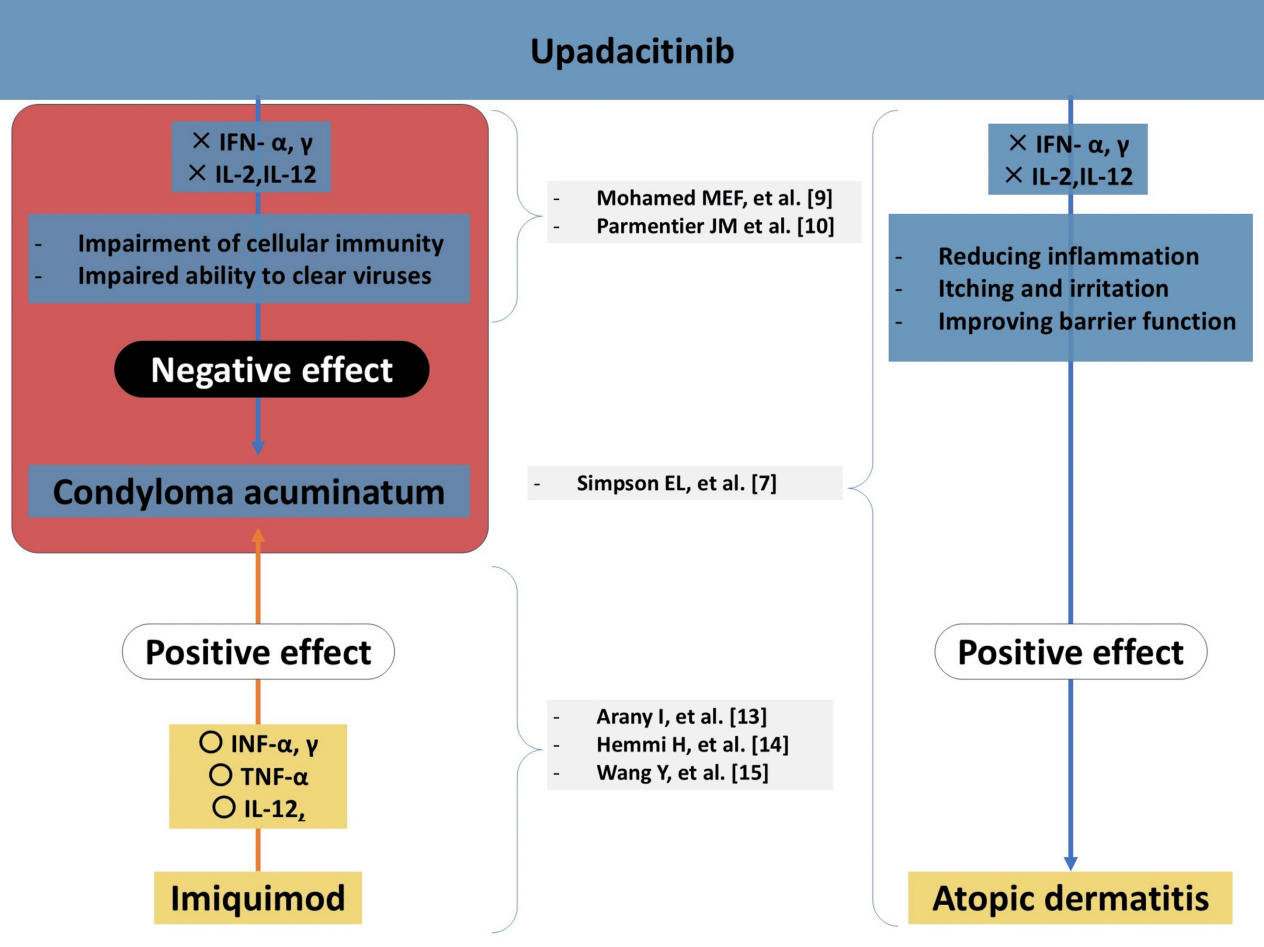
Immunological impact of upadacitinib and imiquimod on viral immunity and skin inflammation. This conceptual illustration depicts the contrasting immunological mechanisms of imiquimod and upadacitinib. Topical imiquimod activates Toll-like receptor 7 (TLR7) signaling in antigen-presenting cells, leading to upregulation of antiviral cytokines such as interferon-α (IFN-α), interferon-γ (IFN-γ), interleukin-12 (IL-12), and tumor necrosis factor-α (TNF-α), which enhance local cell-mediated immunity and facilitate clearance of human papillomavirus (HPV)-infected keratinocytes [13–15]. Conversely, upadacitinib, a selective Janus kinase 1 (JAK1) inhibitor, downregulates downstream signaling of these cytokines (including IFN-α/γ, IL-2, and IL-12) through JAK–STAT inhibition [9,10], thereby suppressing antiviral responses and potentially impairing HPV clearance. This opposing effect may attenuate imiquimod’s immune cascade and contribute to persistence or progression of HPV-related disease in immunosuppressed patients [11]. Original illustration created by the authors (mechanisms referenced in the text). Abbreviations: IFN, interferon; IL, interleukin; TNF, tumor necrosis factor; JAK, Janus kinase; STAT, signal transducer and activator of transcription; TLR, Toll-like receptor

Figure 2 illustrates a conceptual overview of the immunological mechanisms involved. Topical imiquimod activates Toll-like receptor 7 (TLR7) signaling in antigen-presenting cells, leading to production of antiviral cytokines such as interferon-α, interferon-γ, interleukin-12, and tumor necrosis factor-α. In biopsy specimens from anogenital warts, treatment with imiquimod has been shown to upregulate these cytokines and enhance local cellular immunity, thereby facilitating clearance of HPV-infected keratinocytes [13-15].

However, in patients receiving upadacitinib, the therapeutic landscape may shift. Upadacitinib effectively reduces inflammation and improves clinical signs - and by extension barrier function - in atopic dermatitis [7]. However, as a JAK1-selective inhibitor, it attenuates downstream signaling of interferon-α/γ and γ-chain cytokines, such as interleukin-2, and also impacts interleukin-12-related pathways [9,10]. Because topical imiquimod exerts its antiviral effects by triggering TLR7 and inducing a type-1 cytokine milieu (IFN-α/γ, IL-12, TNF) in wart tissue [13-15], JAK1 blockade could blunt the downstream responsiveness to these cytokines, thereby dampening imiquimod’s cascade and the cell-mediated clearance of HPV-infected keratinocytes. Consistent with this mechanism, recent clinical observations have described worsening or recalcitrant HPV-driven warts after initiation of JAK inhibition, including with upadacitinib [11], supporting the possibility that systemic JAK1 inhibition compromises antiviral cellular immunity and facilitates persistence or progression of condyloma acuminatum.

This case highlights the importance of evaluating systemic immune status in patients with recalcitrant HPV-related diseases. Patients undergoing JAK inhibitor therapy, particularly those with recent or multiple sexual partners, should be considered for genital examination and prophylactic HPV vaccination. If new warts emerge or existing ones become refractory during treatment, reassessment of immunosuppressive therapy is warranted.

This case highlights the importance of evaluating systemic immune status in patients with recalcitrant HPV-related diseases. Patients undergoing JAK inhibitor therapy, particularly those with recent or multiple sexual partners, should be considered for genital examination and prophylactic HPV vaccination. If new warts emerge or existing ones become refractory during treatment, reassessment of immunosuppressive therapy is warranted.

## Conclusions

Upadacitinib, a selective JAK1 inhibitor, may impair cellular immunity necessary for HPV clearance, potentially leading to treatment-resistant or persistent condyloma acuminatum. In patients presenting with refractory HPV-related lesions while on immunomodulatory therapy, reassessment of the systemic medication, including drug discontinuation, should be considered to achieve resolution. Moreover, this case illustrates that persistent or rapidly progressive HPV infection can serve as an early clinical signal of excessive immunosuppression in patients receiving JAK inhibitors, reinforcing the need for proactive surveillance in this population.
